# Electroacupuncture efficacy in diabetic polyneuropathy: Study protocol for a double-blinded randomized controlled multicenter clinical trial

**DOI:** 10.1186/s12906-024-04375-8

**Published:** 2024-02-15

**Authors:** María Fernanda Pérez Hernández, Alejandra Calderón Vallejo, Sergio de Jesús Aguilar Castillo, Daniel Cuauhtémoc Gómez Jiménez, Eduardo Rodríguez Guerrero, Fátima Aguilar Morales, Macedonia Guadalupe Moreno Tovar, Miguel Alfredo Zurita Muñóz, Antonio Eduardo Bautista Cortéz, Claudia Camelia Calzada Mendoza, Mónica Ascención De Nova Ocampo, Juan Manuel Ordóñez Rodríguez, Mónica Luz Gómez Esquivel, Alberto García Méndez, Octavio Flores Gil, Víctor Manuel Macías Zaragoza, Gabriela Yanet Cortés Moreno, Citlaltepetl Salinas Lara, Germán Velázquez García, Héctor Iván Saldivar Cerón, Lucia Monserrat Pérez Navarro, Laura Ávila Jiménez, Jaime Héctor Gómez Zamudio, Margarita Díaz Flores, Miguel Cruz López, María Esther Ocharan Hernández, José de Jesús Peralta Romero

**Affiliations:** 1 Unidad de Investigación Médica en Bioquímica, Unidad Médica de Alta Especialidad “Dr. Bernardo Sepúlveda”, Centro Médico Nacional Siglo XXI, IMSS, Mexico City, 06720 Mexico; 2 Sección de Estudios de Posgrado e Investigación, Escuela Superior de Medicina, Instituto Politécnico Nacional, Mexico City, 11340 Mexico; 3 Red MEDICI. Carrera de Médico Cirujano. FES Iztacala, UNAM, Estado de México, State of Mexico, 54090 Mexico; 4 Departamento de Neurología de la Unidad Médica de Alta Especialidad ”Dr. Bernardo Sepúlveda”, Centro Médico Nacional Siglo XXI, IMSS, Mexico City, 06720 Mexico; 5 Departamento de Neurofisiología de la Unidad Médica de Alta Especialidad “Dr. Bernardo Sepúlveda”, Centro Médico Nacional Siglo XXI, IMSS, Mexico City, 06720 Mexico; 6 Sección de Estudios de Posgrado e Investigación, Especialidad en Acupuntura Humana, Escuela Nacional de Medicina y Homeopatía, Instituto Politécnico Nacional, Mexico City, 07320 Mexico; 7 Unidad de Medicina Familiar No. 41, IMSS, Mexico City, 07760 Mexico; 8 Unidad de Medicina Familiar No. 20, IMSS, Mexico City, 07760 Mexico; 9 Unidad de Medicina Familiar No. 44, IMSS, Mexico City, 07320 Mexico; 10 Sección de Estudios de Posgrado e Investigación, Escuela Nacional de Medicina y Homeopatía del Instituto Politécnico Nacional. Maestría en Ciencias en Biomedicina Molecular, Mexico City, 07320 Mexico; 11 Coordinación de Internado y Servicio Social, Escuela Nacional de Medicina y Homeopatía, Instituto Politécnico Nacional, Mexico City, 07320 Mexico; 12 Carrera de Médico Cirujano Facultad de Estudios Superiores Zaragoza, UNAM, Mexico City, 09239 Mexico; 13 Subdirección de Servicios de Salud, Petróleos Mexicanos (PEMEX), Mexico City, 11311 México; 14 Departamento de Neuropatología, Instituto Nacional de Neurología y Neurocirugía, Mexico City, 14269 Mexico; 15 Universidad Intercultural del Estado de México Plantel Tepetlixpa, Tepetlixpa, Estado de México 56880 México; 16 Carrera de Médico Cirujano, Facultad de Estudios Superiores Iztacala, UNAM, Tlalnepantla, State of Mexico 54090 Mexico; 17 Unidad de Biomedicina (UBIMED), Facultad de Estudios Superiores Iztacala, UNAM, Tlalnepantla, State of Mexico 54090 Mexico; 18 Departamento de Nefrología, Hospital General de México “Dr. Eduardo Liceaga”, Mexico City, 06720 Mexico; 19 Coordinación Auxiliar Médica de Investigación en Salud, Jefatura de Servicios de Prestaciones Médicas, Delegación Estatal Morelos, IMSS, Cuernavaca, Morelos 62000 Mexico

**Keywords:** Diabetic neuropathy, Acupuncture, Electroacupuncture, Diabetes, Controlled clinical trial

## Abstract

**Background:**

Diabetic peripheral neuropathy (DPN) is the most common complication of type 2 diabetes mellitus (T2DM); its diagnosis and treatment are based on symptomatic improvement. However, as pharmacological therapy causes multiple adverse effects, the implementation of acupunctural techniques, such as electroacupuncture (EA) has been suggested as an alternative treatment. Nonetheless, there is a lack of scientific evidence, and its mechanisms are still unclear. We present the design and methodology of a new clinical randomized trial, that investigates the effectiveness of EA for the treatment of DPN.

**Methods:**

This study is a four-armed, randomized, controlled, multicenter clinical trial (20-week intervention period, plus 12 weeks of follow-up after concluding intervention). A total of 48 T2DM patients with clinical signs and symptoms of DPN; and electrophysiological signs in the Nerve Conduction Study (NCS); will be treated by acupuncture specialists in outpatient units in Mexico City. Patients will be randomized in a 1:1 ratio to one of the following four groups: (a) short fibre DPN with EA, (b) short fibre DPN with sham EA, (c) axonal DPN with EA and (d) axonal DPN with sham EA treatment. The intervention will consist of 32 sessions, 20 min each, per patient over two cycles of intervention of 8 weeks each and a mid-term rest period of 4 weeks. The primary outcome will be NCS parameters, and secondary outcomes will include DPN-related symptoms and pain by Michigan Neuropathy Screening Instrument (MNSI), Michigan Diabetic Neuropathy Score (MDNS), Dolour Neuropatique Score (DN-4), Semmes-Westein monofilament, Numerical Rating Scale (NRS) for pain assessment, and the 36-item Short Form Health Survey (SF-36). To measure quality of life and improve oxidative stress, the inflammatory response; and genetic expression; will be analysed at the beginning and at the end of treatment.

**Discussion:**

This study will be conducted to compare the efficacy of EA versus sham EA combined with conventional diabetic and neuropathic treatments if needed. EA may improve NCS, neuropathic pain and symptoms, oxidative stress, inflammatory response, and genetic expression, and it could be considered a potential coadjutant treatment for the management of DPN with a possible remyelinating effect.

**Trial registration:**

ClinicalTrials.gov. NCT05521737 Registered on 30 August 2022.

International Clinical Trials Registry Platform (ICTRP) ISRCTN97391213 Registered on 26 September 2022 [2b].

**Supplementary Information:**

The online version contains supplementary material available at 10.1186/s12906-024-04375-8.

## Background

T2DM is a public health problem, according to the International Diabetes Federation (IDF) it was estimated that 537 million people had diabetes in 2021 [1, 2], which occurs nearly in an 80% in low-income and middle-income countries [2], such as Mexico with an estimated direct cost of 50,619 million of pesos per year till 2022 [3]. Nonetheless, only 39% of these patients have an adequate glucose control, which represents a potential risk of developing multiple complications, such as diabetic neuropathy (DN) in its different types of presentation [4].

The most common type is DPN, which accounts for 75% of DN [5]. And during the past ten years (2012–2022), its prevalence has been reported between 7 and 97%, worldwide [6, 7]. Although its exact epidemiological figures are still unknown, according to a recent systematic review and meta-analysis, Mexico has the highest estimated prevalence, compared to other Latin American countries, with a prevalence close to a 69% [8].

DPN is defined as a chronic distal sensory-motor polyneuropathy; attributable to metabolic and microvascular changes caused by glycosylation phenomena, of neuronal proteins, microangiopathy, stimulation of autoantibodies, low-grade chronic inflammation, and nerve capillaries ischemia [9–11].

Hyperglycaemia activates autooxidation processes of glucose, causing nerve demyelination and stimulates monocytes and endothelial cells that enhance this inflammatory response and damage through multiple signaling mechanisms that control DNA transcription; moreover, Nuclear factor kappa B1(NF-kB); and cytokine production, such as tumor necrosis factor alpha (TNF-α), interleukin 1β (IL-1β), interleukin 2 (IL-2), interleukin 6 (IL-6), interleukin 18 (IL-18), and others; and release of reactive oxygen species (ROS), which lead to low-grade chronic inflammation, oxidative stress of mitochondria, and neuronal damage [10, 12–15].

The most common clinical manifestations of DPN are, neuropathic pain, hypoesthesia or anaesthesia, tingling sensation, and weakness, beginning in the toes symmetrically [16]. However, up to 50% of all cases are asymptomatic [17], which increases into a 25%, the risk of pelvic limbs amputation; due to injuries and infections predisposition, that may go unnoticed [9, 11].

According to the American Diabetes Association (ADA), depending on symptoms and clinical examination findings [5]; DPN can be clinically classified as: small-fibre, large-fibre or mixed fibre polyneuropathy (Table 1). Altogether, this must be semi quantified with validated tools, such as MNSI and MDNS, which not only give the clinician the diagnostic suspicion of DPN; but can also estimate its degree of severity. Additionally, pain can be assessed by the DN-4 or the NRS questionnaires, to establish better strategies for pain management [18, 19].

**Table 1:** Symptoms and clinical manifestations of DPN

	Small-fiber neuropathy	Large-fiber neuropathy
**Function**	Nociception and protective sensation	Pressure and balance
**Symptoms**	Pain Burning Electric shocks Stabbing	Numbness Tingling Poor balance
**Clinical tests**	Pinprick Temperature sensation	Ankle reflexes Vibration perception 10 g monofilament Proprioception
**Findings**	Reduced or absent thermal discrimination Reduced or absent Pinprick sensation	Reduced or absent ankle reflexes Reduced or absent vibration perception Reduced or absent 10 g monofilament sensation Reduced or absent proprioception

Even though, clinical examination may provide an estimation of nerve damage severity [20], it can only be confirmed by electrodiagnostic tests, such as NCS, which, despite of being the gold standard, this just evaluates large nerve fibres, whereas small fibres require other types of techniques. However, one of the main limitations of this studies is the operative and economical accessibility, since it is restricted for research purposes or differential diagnosis of atypical neuropathies [16, 18, 19, 21–23].

Due to its diagnostic difficulty, preventive measures have been proposed, focused mainly on glycemic control, modification of triggering and enhancing risk factors, foot care, and vitamin supplementation. But, when symptoms appear, the only option that is left is the instauration of pharmacological treatment with a wide variety of drugs, such as serotonin reuptake inhibitors, calcium channel blockers and tricyclic antidepressants, that, despite of pain relief, they can also cause multiple adverse effects; causing a negative impact on patients’ life quality, and a poor therapeutic adherence or abandonment of treatment; plus, this does not induce a nerve regeneration or remyelination [5, 9, 18, 24, 25].

Therefore, nonpharmacological approaches are needed, and as there is evidence that glycaemic control may not be enough to improve neuropathic symptoms, establishment of other therapies, such as, transcutaneous electrical nerve stimulation, electrical spinal cord stimulation, or acupuncture, is recommended [9, 26].

In recent years, acupuncture; and its variants, such as, laser acupuncture, or EA, have gained popularity and have been broadly used for treating different types of pain. In DPN, it has demonstrated a beneficial effect reducing pain and neuropathic symptoms, that upgrade patient’s life quality; and may even induce an improvement in NCS [26–29]. However, it is not yet clearly described whether these interventions may modify other responses, such as molecular or genetic profiles in humans, since there are few randomized controlled trials (RCTs) [13, 27, 30, 31].

It has been reported that EA produces an analgesic effect by activating cellular and neuronal regeneration pathways, due to the application of a small electric current, apart from needle insertion. Nevertheless, results on symptoms, electrophysiological and some molecular changes, are still not conclusive; because of an incorrect DPN stratification, a small sample size, the lack of a sham control or placebo group and the fulfilment of the ethical research guidelines requirements [32].

For this reason, we have developed a RCT, according to CONSORT [33] and STRICTA [34] guidelines for Good Clinical Practices (GCP) in medical research; guaranteeing a previous adequate diagnosis and DPN stratification by clinical and electrophysiological findings; the implementation of an intervention control group with an accurate randomization, to have an stronger assessment of the clinical, molecular, therapeutic adherence and quality of life, during intervention [6a, 6b].

## Objectives

The aim of this study was, to determine the efficacy, effectiveness, and safety of EA, on the changes of NCS electrophysiological parameters, in patients with DPN; compared to a sham EA group, during and after intervention. With the assessment of other secondary objectives, as follows:

1. Semiquantitative evaluation of neuropathic symptoms diminishment, by the, MNSI, MDNS, DN-4 and NRS questionnaires.
2. Estimation of the impact of interventions on life quality, by the SF-36 questionnaire.
3. Assessment of the modification of molecular variables, as part of pathophysiology:
  - Oxidative stress.
  - Inflammatory response.
  - Gene expression.
4. Therapeutic adherence monitoring, to establish possible interventional adverse events [7].

## Trial design

This is a, four-armed, randomized, double-blinded, multicenter, placebo-controlled trial; to evaluate EA effectiveness on, clinical, electrophysiological and molecular changes; along with its safety for DPN treatment; in an intention to treat (ITT) analysis, attached to real life [8].

## Methods: Participants, interventions and outcomes

### Study setting

This study will be conducted at the Unidad de Investigación Médica en Bioquímica (UIMB), Neurology and Neurophysiology Departments of the Unidad Médica de Alta Especialidad (UMAE) “Dr. Bernardo Sepúlveda”, Centro Médico Nacional (CMN) Siglo XXI, Instituto Mexicano del Seguro Social (IMSS), Mexico City.

With the recruitment centres at the family care units, UMF 20, 44 and 41, of the IMSS, Mexico City. The intervention center at the Escuela Nacional de Medicina y Homeopatía (ENMyH), Instituto Politécnico Nacional (IPN), Mexico. The external collaborators for database management at, Facultad de Estudios Superiores (FES) Iztacala, Universidad Nacional Autónoma de México (UNAM), State of Mexico; FES Zaragoza, UNAM, Mexico City; Instituto Nacional de Neurología y Neurocirugía “Manuel Velasco Suárez”, Mexico City; and Universidad Intercultural del Estado de México, Tepetlixpa, State of Mexico. And the statistical analysis centers at, Delegación Estatal de Investigación Morelos, IMSS, Cuernavaca, Morelos; and Hospital General de México (HGM) Dr. Eduardo Liceaga, Mexico City [9].

The protocol for this trial is reported based on the Standard Protocol Items: Recommendations for Interventional Trials (SPIRIT) 2013 Checklist: defining standard protocol items for clinical trials (Additional files 1 and 2).

This trial was approved on May 26th, 2020, by the Scientific Research Committee IMSS (R-2020–785-070,), Research Ethics Committee of the Comisión Nacional de Bioética (CONBIOÉTICA) (CONBIOÉTICA: 09CEI00920160601), and the Comisión Federal para la Protección Contra Riesgos Sanitarios (COFEPRIS) Research Committee (CI: 17 CI 09 015 006). And international trial registrations: ClinicalTrials.gov Identifier: NCT05521737. Registered prospectively, in August 30th, 2022 https://classic.clinicaltrials.gov/ct2/show/NCT05521737?term=electroacupuncture&cond=diabetic+neuropathy&draw=2&rank=2 and International Clinical Trials Registry ISRCTN97391213. Registered on September 12th, 2022 – prospectively registered, https://www.isrctn.com/ISRCTN97391213 [2a].

## Patients and recruitment

### Sample size

According to Wang; et al*. *[31], we calculated a sample size with the free-use software G*Power 3.1, by a two-sided T test for dependent samples, based on mean and standard deviation changes before and after treatment of peroneal motor nerve conduction velocity in EA group (40.15 ± 3.06 m/s, and 42.97 ± 2.74 m/s, respectively). With an 80% statistical, a 5% alpha error, and a possible 20% loss.

Resulting on a sample size of 12 patients, per interventional group, which represents a total sample size of 48 patients, stratified into two severity groups plus two interventional groups each. That is, 24 small fibres, and 24 axonal DPN; allocated into two interventional groups (12 subjects for EA and 12 subjects for sham EA, per DPN severity group). In this context, we will recruit approximately 200 patients at screening, due to inclusion criteria [14].

## Eligibility criteria

### Inclusion criteria

1. Patients with T2DM.
2. Reached age between 40 and 75 years old.
3. Both sexes (men and women).
4. Reported neuropathic symptoms.
5. Confirmed clinical diagnosis of DPN by physical examination.
6. Electrophysiological diagnosis of DPN in its different types of severity (small fibres or axonal DPN).
7. Understanding and signing the informed consent form.

### Exclusion criteria

1. Neuropathy or chronic pain caused by other conditions, than diabetes.
2. Presence of serious medical conditions, including, kidney diseases, heart diseases, pulmonary diseases, liver diseases or contagious diseases, neurological diseases, treatment with cancer drugs, systemic autoimmune diseases, hematological disorders, HIV diagnosis, and others.
3. Pregnant or breast-feeding women.
4. Previous history of spinal or hip trauma, fracture, or replacement surgery.
5. Ulceration of the lower extremities or previous amputations.
6. Treatment with acupuncture or moxibustion in the last 6 months, previous to intervention.
7. Patients with pacemarkers.
8. Unable to complete measurement questionnaires or physical examinations.
9. Recruited in other clinical trials simultaneously.

### Withdrawal criteria

1. Development of serious adverse events during intervention.
2. Withdrawal demanded by the participant.
3. Patients who become pregnant during interventional period [10].

## Recruitment

To achieve the target sample size, patients will be captured at the different primary health care units, UMF 20, UMF 41 and UMF 44, through a telephonic invitation to a DPN prevention program to the potential candidates obtained through the databases of the recruitment centres; in which we will offer a complete neurological evaluation, complemented with the electrophysiological if needed, as well as medical advice for prevention or control of DPN and other diabetes complications [15].

## Concealment

Before randomization, a clinical physician will inform patients about the different procedures contemplated in the study, along with the possible benefits or side effects. After that, written consent will be obtained from each participant, previous to the baseline evaluation (Additional file 3) [16b, 26a].

## Allocation sequence

Individual patient randomization will be performed at a 1:1: ratio, into the four study arms of group intervention; using a random-numbers table by an independent statistician, who will not be involved in the statistical analysis of the trial and will keep the allocation sequence separately [16a].

## Allocation implementation

Treatment allocation will only be revealed to acupuncturists out of patients’ sight, to ensure blinding, and they will be discouraged from discussing treatments or previous results with patients, to reduce the risk of observer bias [16c].

## Masking

Due to the intervention characteristics, acupuncturists will not be blinded. However, patients and data analysis statisticians will be blinded to group assignment. Treatment will be unrevealed to patients until all of the outcomes and statistical analysis are completed. During the study, patients will receive either of the interventions blindfolded, placing a bandage over their eyes, so that they cannot see the type of therapy that will be applied [17a].

## Procedure for unblinding if needed

Blinding will only be broken if statistically significant differences are obtained in the interim comparative pre-analysis. Therefore, we might assume the need to grant the benefit to all patients once the study is completed in its entirety for all participants [17b].

## Study intervention

### Phases of the study

**Fig. 1:**
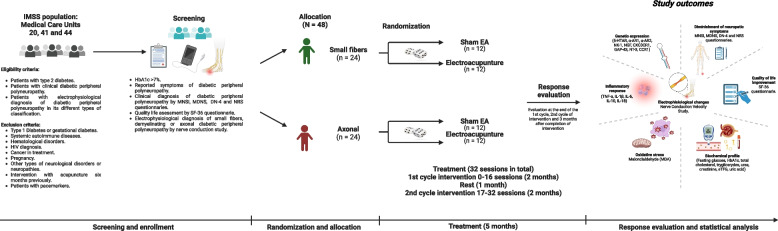
Protocol graphical abstract

The overall Study for each patient consists of four phases, described below and in Fig. 1 and Additional file 4.

#### Phase I. Testing

Patients are captured on the database of the Primary Health Care Hospital, Sistema de Información de Medicina Familiar (SIM). After this, they are phone called to introduce them the protocol and make them the invitation to participate. If patients are interested in participating, a brief short form with the main eligibility criteria is applied. Once they meet the necessary eligibility criteria to enter the study, an appointment at its family care unit is scheduled, in order to obtain the written informed consent form, to assess the MNSI, MDNS, DN-4, NRS, and SF-36 questionnaires; additionally, to a physical neurological clinical examination by a general practitioner, that will establish the clinical diagnosis of DPN. After that, the clinical diagnosis will be corroborated by a second neurological evaluation in charge of a neurology specialist at the neurology department of the UMAE “Dr. Bernardo Sepúlveda” at CMN Siglo XXI.

#### Phase II. Screening

For patients with a confirmed clinical and neurological diagnosis of DPN, an electrophysiological evaluation by NCS will be performed; so that DPN severity can be assessed, and to dismiss other types of neuropathies. According to this, patients could be stratified into two main groups: short fibres and axonal DPN.

#### Phase III. Randomization and allocation

Once DPN severity is assessed, patients will be randomized and allocated to either of the interventions (EA or sham EA). However, before starting the intervention, patients will be called to schedule a blood sample collection appointment at its UMF, for biochemical and molecular test outcomes.

#### Phase IV. Intervention

Subsequently, patients will be contacted by acupuncturist technicians by phone call, to schedule their first interventional session. All groups will receive a total of 32 sessions of treatment, over 37 weeks; divided into two cycles of intervention (EA or sham EA). That is, 16 sessions each, over 8 weeks, with 2 sessions a week, and a rest of 4 weeks between them. Including a postintervention follow-up, extended three months after concluding treatment.

If patients cannot attend any of their scheduled appointments, they will be asked to reschedule it for another day in the same week.

Each patient will receive routine health care for diabetes and comorbidities control, during the entire study. Any changes in pharmacological treatment (including date of administration modifications, types, and dosage) will be recorded in detail.

At the end of each cycle of intervention, clinical, electrophysiological, biochemical, and molecular parameters will be reassessed to compare changes during intervention.

#### Phase IV. Follow-up

After the 32 sessions have been completed, participants will have a three-month additional follow-up period. During this term, no EA or sham EA intervention will be given. As well as any other type of acupuncture treatment will not be permitted.

Once follow-up has finished, clinical, electrophysiological, biochemical, and molecular variables will be re-evaluated to assess the intervention efficacy over time [13].

## Interventions description

### Electroacupuncture and sham EA device

**Fig. 2:**
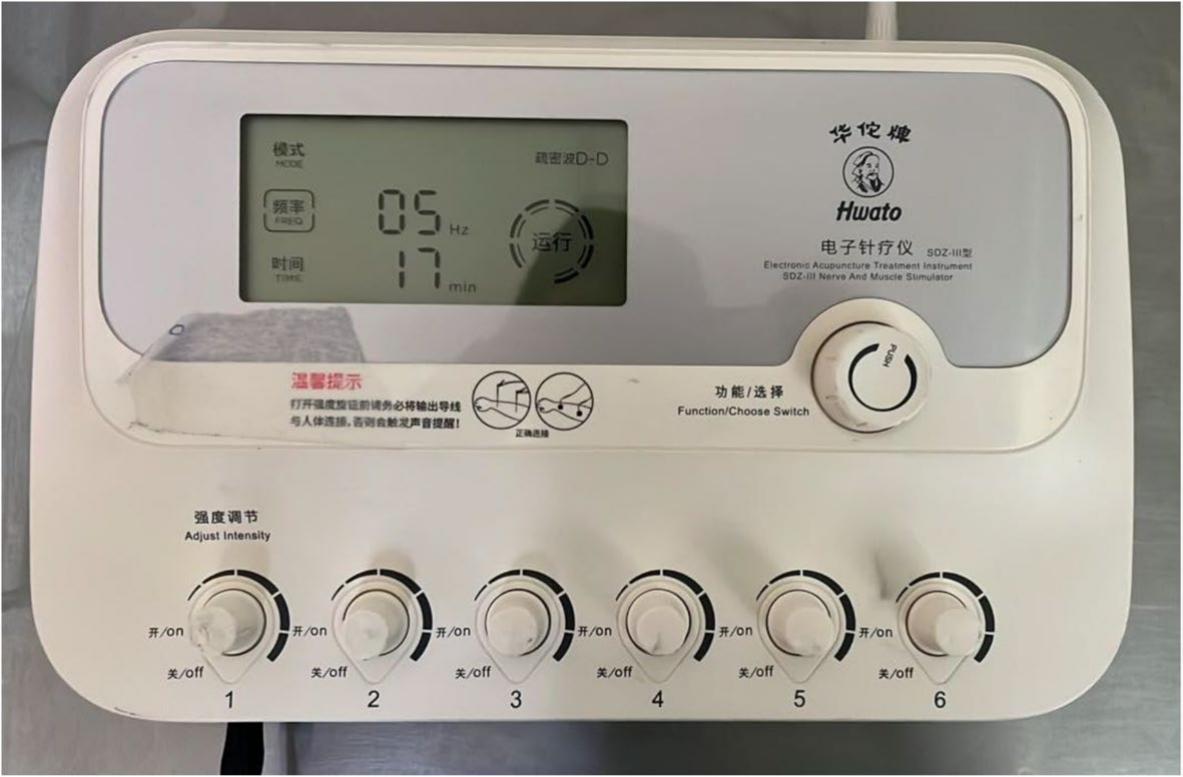
Chinese HWATO-SDZ III low-frequency pulse electronic acupuncture stimulator machine

Chinese HWATO-SDZ III Low-Frequency (1 to 100 Hz) Pulse Electronic Acupuncture Stimulator Machine, with Large Screen Display, 6 channels, and adjustable output power (0.3 to 10 V at 250 ohms). Plus 3 available waveforms: continuous, intermittent and both alternating (Fig. 2). Both sham EA and EA devices will be identical in appearance, weight, sound, and operational procedure; but without electronic pulse stimulation output, for sham EA groups.

### Electroacupuncture

**Fig. 3:**
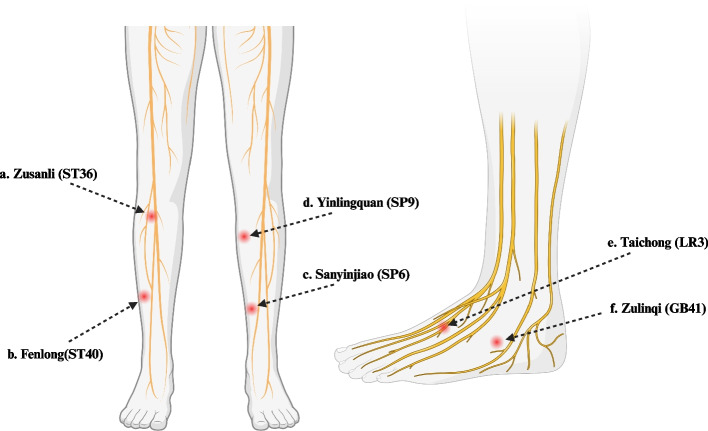
Acupoints location

Patients will lie down in supine position, with their legs entirely exposed above the knee. The stimulated acupunctural points will be Zusanli (E36), Fenlong (E40), Yinlingquan (B9), Sanyinjiao (B6), Taichong (H3) and Zulinqi (VB41) [35–37] (Table 2 and Fig. 3). Acupuncture S-Type needles (30Gx40mm) will be inserted, with the application of a dense/disperse wave of 2 Hz, for 20 min in the different acupoints at the same time. The distance between the tips and the skin is approximately 1.5 and 2 cm; until Deqi sensation is stimulated [38, 39].

**Table 2:** Acupoints

Acupoint	Location	Stimulated Nerve
**Zusanli (ST36)**	Located at the tibialis anterior muscle four finger breadths of subject below the kneecap and one finger breadth of subject lateral from the anterior crest of the tibia	Common Peroneal Nerve
**Fenlong (ST40)**	Eight finger breadths above the maximum prominence of the external malleolus, and 1,5 finger breadths laterally towards the anterior border of the tibia	Branches of Peroneal Nerve
**Sanyinjiao (SP6)**	Three finger breadths above the maximum protuberance of the malleolus medial, dorsally to medial tibial border	Tibial Nerve
**Yinlingquan (SP9)**	One or two finger breadths above the posteroinferior borderline of the internal tibial condyle	Saphenus Nerve
**Taichong (LR3)**	0.5 to 0.8 finger breadths laterally to the dorsum of the foot, in the distal depression of the angle proximal between the 1st and 2nd metatarsal	Deep Peroneal Nerve
**Zulinqi (GB41)**	0.5 to 0.8 finger breadths laterally to the dorsum of the foot, at the proximal angle between the 4th and 5th metatarsals, in the lateral depression of the tendon of the m. extensor 5th finger longus	Branches of Tibial Nerve

### Sham electroacupuncture

**Fig. 4:**
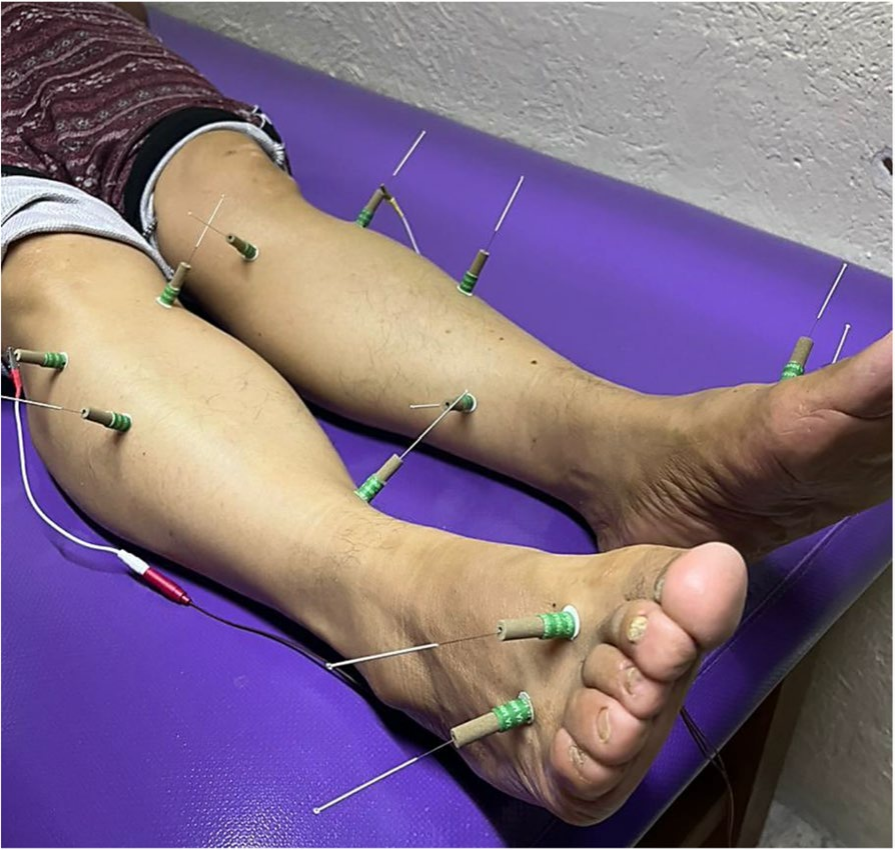
Sham electroacupuncture device

Sham EA will be applied using a nonpuncture device, made out of a Chinese Mugwort Tube Moxa Cone Self Stick, which prevents needle penetration; inserted at the same acupoints as the EA group. The device procedure will be identical as EA intervention, but without electrical stimulation, and needle manipulation, for Deqi stimulation (Fig. 4) [11a].

## Criteria for discontinuing modification of the allocated interventions

1. Patients demanded drop out.
2. Worsening or improvement of neuropathic symptoms.
3. Serious side effects that put patients’ health at risk [11b]

## Strategies to improve adherence to interventions

For treatment adherence assessment, a specific physician of the UIMB, and Universidad Intercultural del Estado de México, Tepetlixpa, will call them once every two weeks during intervention term, to verify patients’ therapy attendance, and to identify possible interventional side effects [11c].

## Relevant concomitant care permitted or prohibited during the trial

Patients are allowed to receive any modifications for blood sugar, DPN pain, or chronic comorbidity control. Usage of complementary medicine such as traditional Chinese herbs or acupuncture variants; and the uptake of new physiotherapeutic procedures are not permitted [11d].

## Provisions for post-trial care

Patients who complete the whole intervention, including the follow-up period, will receive a detailed record report, comparing their baseline clinical status, with the rest of the measurements, and medical advice to improve their adherence to treatment, and reduce their risk of further complications [30].

## Outcomes

All of the outcomes will be measured at the four different timepoints (baseline, at the 2nd and 3rd visit after treatment cycles are completed; and at follow-up (three months after the end of the intervention), to evaluate changes with intervention (EA or sham EA).

### Primary outcome

#### Nerve conduction study

NCS will be executed by a neurophysiologist specialist using the VIKING LIFE-SAVING EQUIPMENT; to determine the motor and sensitive NCS of the common peroneal, sural, and tibial nerves.

### Secondary outcomes

#### MNSI

It will be assessed by the application of a brief dichotomic questionnaire of neuropathic symptoms, in which “yes” responses to items 1–3, 5–6, 8–9, 11–12, and 14–15, are each counted as one point. A “no” response on items 7 and 13 counts as 1 point. Items 4 and 10 are not included in the scoring. A total score of 7 points establishes a clinical diagnosis of DPN.

Then, it is completed by a physical examination, executed by a health professional, of both lower extremities: foot appearance and ulceration (0 or 1 for normal and abnormal, respectively, ankle reflex (0 = normal, 0.5 = reenforced, or 1 = absent), vibration test and monofilament examination (0 = normal, 0.5 = weakened, and 1= absent). A total score of 2.5 points will provide a clinical diagnosis of DPN [40].

#### MDNS

Measured with three main indicators, perception of vibration, 10 gr. monofilament and pin prick, each qualified as: 0 = normal, 1 = diminished, or 2 = absent.

In addition to the muscular strength of the toes, and ankle dorsiflexion, quizzed as: 0 normal, 1 = diminished, 2 = severe, or 3 = absent.

The evaluation of bicipital, tricipital, quadriceps and Achilles muscle reflexes, each qualified as: 0 = present, 1 = present with effort, or 2 = absent.

A total of 0 to 6 points will be considered as no neuropathy, a score between 7 and 12 points as mild neuropathy, 13 to 29 points as moderate neuropathy, and severe neuropathy if the total score is between 30 and 46 points [41].

#### Douleur Neuropathique en 4 Questions (DN-4)

A 10-symptom item questionnaire, about the presence of neuropathic symptoms such as, burning, cold, electric shocks, tingling, numbness, itching, hypoesthesia, and pain. A score of 4 or more, out of 10, is suggestive of neuropathic pain [18, 42].

#### NRS

A single 11-point numeric scale for pain assessment, where patients are asked to rate their current pain and during the past 7 days, on a scale from 0 to 10, with 0 indicating no pain and 10 reflecting the worst possible pain of their lives. NRS is often conducted as a scale interpreted as follows: 0 = no pain, 1–3 = mild pain, 4–6 = moderate pain, and 7–10 = severe pain [43].

#### Quality life (SF-36)

A validated tool for quality life measurement, that consists of 36 items, divided into eight different areas: physical functioning, 10 items (questions 3-12); role limitations due to physical health, 4 items (questions 13-16); role limitations due to emotional problems, 3 items (questions 17-19); energy or fatigue, 4 items (questions 23, 27, 29, and 31); emotional well-being, 5 items (questions 24-26, 28, and 30); social functioning, 2 items (questions 20 and 32); pain, 2 items (questions 21 and 22); and general health, 5 items (questions 1, 33-36). Each item is scored on a 0 to 100 range into a 5-grade scale, 100, 75, 50, 25, and 0; where a higher score indicates a better health on each dimension. So, a 100 score represents a perfect health, while a 0 score indicates the worst possible health [44].

#### Oxidative stress

Determined by quantifying plasma concentrations of the lipoperoxidation subproduct; malondialdehyde (MDA), according to Yagi, et al. technique, where plasma MDA reacts with thiobarbituric acid (TBA), to form the cholorimetric complex “MDA-TBA”, which will be measured by spectrophotometry at 530 nm [45].

#### Inflammatory response

Changes in serum concentrations of proinflammatory (IL-6, IL-1β, TNF-α and IL-18) and anti-inflammatory (IL-10) cytokines, determined by flow cytometry quantitative ELISA, with the Biolegend kit, LEGENDplex TM Human Inflammation Panel 1 Detection Antibodies.

#### Genetic expression

Measured by mRNA expression of the 5-HT1AR (serotonin 1A receptor), NK-1 (neurokinin 1), α-AR1 and α-AR2 (α-adrenoreceptors 1 and 2), NGF (nerve growth factor), CX3CR1 (CX3C motif chemokine receptor 1), GAP-43 (Growth associated protein 43), NT3 (Neurotrophin), and CCR1 (Chemokine receptor 1) genes, quantified by real-time qPCR. Probes of the Human Universal Probe Library; and a thermal cycler Techne Prime Pro-48 Real-Time qPCR; with a reaction mixture type TaqMan; from the brand "SensiFAST Probe No-ROX kit/Bioline". The sequences of primer oligos (sense and antisense) will be designed with Probe software Finder version 2.45 (http://www.universalprobelibrary.com).

#### Safety

Adverse effect reports will be monitored during the entire protocol, by a specific physician responsible for calling patients, every two weeks to verify their health status, and it will be recorded in a specific format that will be sent to COFEPRIS annually. Just in case a major adverse effect is reported, such as death, permanent damage, or requirement of hospitalization, it will be communicated in real-time.

### Confounding variables

#### Anthropometry and biochemical measurements

Anthropometric variables as, height, weight and body mass index (BMI), waist and hip circumference; and blood pressure, will be measured at each timepoint with the Seca 213 Portable Stadiometer, Body Composition Analyser BC-418, Seca 203 measuring tape, and the OMRON HEM-705CPINT blood pressure monitor, respectively.

Biochemical parameters of fasting glucose, HbA1c%, total cholesterol, triglycerides, uric acid, creatinine, urea, and BUN; will be assessed at each timepoint with AU480 clinical chemistry equipment; from Coulter Beckman; using a photometric technique to determine the concentrations of the analytes, which will be validated through the Roche Lab core System [12].

### Plans for the collection, laboratory evaluation, and storage of biological specimens for genetic or molecular analysis in this trial/future use

Additionally, blood sample specimens will be collected to form total blood, serum, and plasma sample biobanks, for further molecular, genetic, and other analyses. These samples will be sheltered at the main research centre, UIMB [33].

### Data collection

For the measurement of outcomes, a team will be trained for each of these results, that is, for neurological examination, supervised by a neurologist, for taking laboratories and processing biochemical and molecular tests, supervised by a pharmacobiological chemist, while electrophysiological studies will be carried out by a neurophysiologist, as previously described. Data results will be obtained from the physical questionnaires, and electrophysiological, biochemical, and molecular reports, which will later be digitalized and entered into an online platform by a clinical coordination department, in charge of organizing and supervising the management of this documents. After this, an independent researcher will create an online database on the same platform and create a second digital back up on an independent computer owned by the UIMB and the principal investigator JJPR. [18a].

### Data retention

All research documents, including patients’ paper files and electronic documents, and databases will be preserved for at least 7 years after the study completion date. Just the medical support team, the database curation supervisors and principal investigator, will have free access to these reports [18b].

### Data management

As previously described, an independent researcher at UIMB, not involved in patient recruitment, intervention or statistical analysis; will enter each patient’s information into a database, on a particular computer exclusively used for its electronic storage, apart from an online backup. Additionally, it will be supervised by a second independent researcher and third researcher at different institutions (HGM and Delegación Estatal de Investigación Morelos, IMSS), to ensure the accuracy of the data [19].

### Statistical analysis

Collected variables will be integrated into a Microsoft Access Program database; then it will be analysed in SPSS (Statistical Product and Service Solutions) program, version 25, for Windows.

Descriptive analysis of collected data will be carried out using simple and absolute frequencies, as well as measures of central tendency and dispersion. Additionally, to the application of the Shapiro–Wilk test, for data distribution assessment of quantitative variables, such as, MNSI, MDNS, DN-4, NRS and SF-36 total scores questionnaires, electrophysiological variables of the NCS (sensitive and motor latency, amplitude, and nerve velocity of sural, tibial and peroneal nerves), and biochemical and molecular parameters.

Changes throughout the intervention of quantitative variable for each of the four interventional groups at their different evaluation timepoints (baseline, first and second visit, and follow-up), will be analysed by an ANOVA paired sample test; and to compare differences between interventions (EA vs sham EA), a T test for independent groups will be performed at each timepoint.

For the analysis of the reported symptoms of diabetic neuropathy during intervention, a comparison will be made using Cochran’s Q test for each interventional group; and to assess the difference in distribution of independent groups at each study timepoint, an exact Fisher’s test will be applied.

Finally, multiple linear regression analysis will be performed, to establish the effect size of receiving EA, adjusted by other independent variables that might influence the study outcomes, such as HbA1c%, and administration of pharmacological treatment for neuropathic pain.

For all tests, a value of *p* < 0.05 will be considered statistically significant [20a].

### Missing data analyses

The analysis will be performed with the full analysis set (FAS) based on an ITT analysis. Missing values will not be replaced. [20c].

### Additional analyses

Further analyses will be regarded as explorative (with possible adjustment and multiple imputation for better results) [20b].

### Interim analyses

Interim analyses are contemplated every four months, and just the data monitoring committee and the principal investigator, JJPR, will have access to the preliminary results, and he will make the final decision to finish the trial [21b].

### Harms

Safety will be monitored by an independent clinical expert, with access to unblinded data, in addition to monthly scheduled meetings to report each patient’s clinical status, and to monitor treatment efficacy and safety, during trial development [22].

### Auditing

The principal investigator, JJPR, will prepare an annual report that will be delivered to the corresponding financing institutions [23].

## Roles and responsibilities

### Funder

Funders will only be responsible for granting financial support. Any changes on study design, reports, publications, or further activities are under consent of the principal investigator, JJPR [5c].

### Research committees

#### Medical monitoring committee

Conformed by the physicians at the recruitment and research centres. Who will supervise and secure the accomplishment of the regulatory requirements to conduct the protocol.

#### Safety review committee

An independent committee of allocation, intervention, data, or statistical analysis, who will monitor the clinical trial progress, and review safety and effectiveness of interventions while the trial is still ongoing.

#### Data monitoring committee

It will be independent from the funders and without any competing interests, conformed by MFPH at UIMB, LMPN at HGM “Dr. Eduardo Liceaga”, and LAJ at Delegación Estatal de Investigación Morelos, IMSS, Cuernavaca, in charge of doing interim comparison analyses, to make recommendations on modifying, continuing or finishing the trial [21a].

#### Clinical endpoint committee

Consisting of the data monitoring committee members, as well as the acupuncturists, neurologists and neurophysiology supervisors; and the principal investigator; who will review of the source data and medical events according to the estimated outcomes.

#### Steering committee

It will review the protocol, documents, or individual data, to provide recommendations to ensure the project success at UIMB [5d].

## Competing interests

None of the research groups received a study grant for conducting this clinical study, travel expenses and honoraria for a presentation of the scientific content, honorary fees for teaching acupuncture classes or lecture fees [28].

## Ethics approval and consent to participate

Ethical approval was obtained on May 26^th^, 2020, by the Scientific Research Committee (R-2020–785-070, IMSS), Research Ethics Committee (CONBIOÉTICA: 09CEI00920160601), and the COFEPRIS Research Committee (CI: 17 CI 09 015 006). Trial registration: ClinicalTrials.gov Identifier: NCT05521737. Registered 30 August 2022—prospectively registered, https://classic.clinicaltrials.gov/ct2/show/NCT05521737?term=electroacupuncture&cond=diabetic+neuropathy&draw=2&rank=2; and ICTRP: ISRCTN97391213, registered 12 September 2022 – prospectively registered https://trialsearch.who.int/Trial2.aspx?TrialID=NCT05521737.

According to the General Health Law on Health Research published in the Official Gazette of the Federation on February 7, 1984, this study is classified as an investigation with greater than the minimum risk, as it is a double-blind randomized clinical trial.

Its creation and development attached to the Declaration of Helsinki on Ethical Principles for Medical Research on Human Subjects and the GCP Guidelines [24].

## Informed consent

Therefore, the authorization of the participants through an informed consent form is essential. It will explain the objective of the study and significance, procedures, benefits and potential risks, confidentiality, and the names of those responsible for the study and clarify the autonomy and freedom they must participate. In addition, as part of the ethical considerations, it will be reported that 20 mL of peripheral venous blood will be extracted, which does not endanger participants’ health and will be treated carefully according to NOM-087-ECOL-SSA1-2002, Secretaría de Salud de Mexico [26b].

## Confidentiality

It will be clarified that the information provided will be confidential, and only the medical staff and researchers will have access to the information. The results published or presented at conferences will not reveal the identity of the patient. All data obtained will be the property of the UIMB, without conflict of interest [27].

## Protocol re-approvals and amendments

Re-approvals will be made annually, as set out by the institution's research and ethics committees. While amendments will depend on the necessary methodological changes for the development of the protocol [25].

## Consent for publication

### Dissemination policy: trial results

The medical support team and acupuncturists will give a weekly report to the principal investigator. Principal investigator will submit an annual report of the adverse effects reported to the ethics committee and COFEPRIS. Participants will only receive the full report after all stages of the study have been completed, or in case that they must withdraw the study [31a].

### Dissemination policy: reproducible research

Detailed information of the study is open access at the international registration platforms, clinicaltrials.gov and the ICTRP platform. Full access to data and statistical code will be considered by the principal investigator. If reviewers or readers have any questions regarding our published data, they can contact the corresponding author via email [31c].

## Availability of data and materials

The principal investigator PhD José de Jesús Peralta Romero; has unlimited access to the final trial dataset. Any data needed to support the protocol can be supplied upon request, by the principal investigator via email at drjperalta@hotmail.com or drjjperalta@gmail.com [29].

## Funding

At the moment, this work is supported by grants from Instituto Mexicano del Seguro Social (IMSS Prioritarios R-2020-785-070); SECTEI (SECTEI/132/2023), and CONACHYT (CF-2023-G-1314) [4].

## Trial status

This trial is currently recruiting patients. Until now, we have made two re-approvals, dated on July 9th, 2021; and November 18th, 2022; and we have requested a last re-approval on January 11th, 2024. This is the second version of the protocol, approved on the 17th of November 2022. Recruitment began in November 2021, and the anticipated recruitment end date is June; 2024 [3].

## Discussion

DPN is the most common complication of diabetes, causing higher rates of disability worldwide due to its difficult diagnosis and management. Even though, the main problem of this condition is poor glycemic control, pharmacological treatment has a potential risk of developing multiple secondary events. So, it is important to establish different strategies for neuropathic pain and symptom management, such as acupuncture and its variants, such as EA, which have been described to benefit patients in pain relief, despite of glycaemic control, DPN severity, and other underlying patients’ conditions, with much less or even without any risk of presenting adverse effects, tolerance, dependence, addiction, or abuse as drugs.

Furthermore, it is believed that EA might have a remyelinating effect, as it has been reported, that, both motor and sensitive nerve conduction velocity, of sural and tibial nerves, increase significantly over time [29, 31]. Additionally, some authors have described in preclinical studies that acupoints stimulation is able to activate an anti-inflammatory cellular response by vagus nerve stimulation, and production of acetylcholine, which inhibits transcription factors for proinflammatory cytokine production [40, 46].

However, as most of them are set in animal models; and those that have been performed in humans, lack randomization, blinding, a control group, a larger sample and time of intervention, further studies are needed to assess in a more objective manner the effect of EA.

Therefore, we chose a total intervention of 16 weeks with a middle-rest term of one month to prevent tachyphylaxis phenomena secondary to the release of multiple vasoactive mediators that may induce dangerous responses when administered constantly for a longer period [47].

On the other hand, we consider NCS changes as the main outcome for our study, as this is the gold standard for its highest sensitivity, so we will not only have the clinical diagnosis by the semiquantitative questionnaire MNSI, MDNS, DN-4, and NRS scores; we will stratify DPN according to electrophysiological findings. Additionally, other objective measures will be assessed, including oxidative stress, inflammation, and genetic expression, which is not conclusive or has not been well studied in humans. All of this is in consideration of patients’ quality of life evaluation by the SF-36 questionnaire, which makes this clinical trial a translational research project not only to try to elucidate EA mechanisms in DPN; but also, to seek the direct application of the obtained results for direct patient benefit.

### Supplementary Information

**Additional file 1. **SPIRIT figure of the phases of the trial and data collection time points.

**Additional file 2. **SPIRIT Checklist for Trials.

**Additional file 3.** Informed Consent form.

**Additional file 4: Supplementary figure 2.** protocol consort diagram.

## Data Availability

Data will not be openly shared, just the principal investigator PhD José de Jesús Peralta Romero; will have unlimited access to the final trial dataset. Any data needed to support the protocol can be supplied upon request, by the principal investigator via email at drjperalta@hotmail.com or drjjperalta@gmail.com.

## Abbreviations

DPN: Diabetic polyneuropathy
T2DM: Type 2 Diabetes Mellitus
EA: Electroacupuncture
NCS: Nerve Conduction Study
MNSI: Michigan Neuropathy Screening Instrument
MDNS: Michigan Diabetic Neuropathy Score
DN-4: Douleur Neuropathique en 4 Questions
NRS: Numeric Pain Rating Scale
SF-36: 36-Item Short Form Health Survey questionnaire
ICTRP: International Clinical Trials Registry Platform
IDF: International Diabetes Federation
DN: Diabetic Neuropathy
DNA: Deoxyribonucleic acid
NF-kB: Nuclear factor kappa B1
TNF-α: Tumor Necrosis Factor Alpha
IL-1β: Interleukin 1β
IL-2: Interleukin 2
IL-6: Interleukin 6
IL-18: Interleukin 18
ROS: Reactive oxygen species
ADA: American Diabetes Association
RCTs: Randomized Controlled Trials
CONSORT: Consolidated Standards of Reporting Trials
STRICTA: STandards for Reporting Interventions in Clinical Trials of Acupuncture
GCP: Good Clinical Practices
ITT: Intention to Treat
UIMB: Unidad de Investigación Médica en Bioquímica
UMAE: Unidad Médica de Alta Especialidad
CMN: Centro Médico Nacional
IMSS: Instituto Mexicano del Seguro Social
UMF: Unidad de Medicina Familiar
ENMyH: Escuela Nacional de Medicina y Homeopatía
IPN: Instituto Politécnico Nacional
FES: Facultad de Estudios Superiores
UNAM: Universidad Nacional Autónoma de México
HGM: Hospital General de México
SPIRIT: Standard Protocol Items: Recommendations for Interventional Trials
CONBIOÉTICA: Comisión Nacional de Bioética
COFEPRIS: Comisión Federal para la Protección Contra Riesgos Sanitarios
HIV: Human Immunodeficiency Virus
SIM: Sistema de Información de Medicina Familiar
MDA: Malondialdehyde
TBA: Thiobarbituric acid
IL-10: Interleukin 10
ELISA: Enzyme-linked immunosorbent assay
5-HT1AR: Serotonin 1A receptor
NK-1: Neurokinin 1
α-AR1: α-Adrenoreceptor 1
α-AR2: α-Adrenoreceptor 2
NGF: Nerve growth factor
CX3CR1: CX3C motif chemokine receptor 1
GAP-43: Growth associated protein 43
NT3: Neurotrophin
CCR1: Chemoline receptor 1
HbA1c%: Glycated hemoglobin
BUN: Blood urea nitrogen
FAS: Full analysis set
SECTEI: Secretaría de Educación, Ciencia, Tecnología e Innovación
CONACHYT: Consejo Nacional de Humanidades, Ciencias y Tecnologías
