# Supplementary material for: Electroacupuncture efficacy in diabetic polyneuropathy: Study protocol for a double-blinded randomized controlled multicenter clinical trial
Source: BMC Complement Med Ther. 2024 Feb 15;24:90. doi: 10.1186/s12906-024-04375-8 (PMC10868023; doi:10.1186/s12906-024-04375-8)
Supplement: Supplementary file 1 — Additional file 1. SPIRIT figure of the phases of the trial and data collection time points. [file 12906_2024_4375_MOESM1_ESM.docx]

Additional file 1. SPIRIT figure of the phases of the trial and data collection time points.

# STUDY PERIOD

Enrolment

Allocation

Intervention (Post-allocation)

Efficacy

# TIMEPOINT ENROLMENT:

Pre- randomization

t0

Randomization t0

1st visit (Baseline)

t0

1st cycle of intervention

Week 1-8

**2nd visit**

Week 9

Rest

Week 10-14

2nd cycle of intervention

Week 15-23

**3rd visit**

Week 24

Follow-up

+3 months without intervention

**4th visit**

Week 37

Eligibility screen X

Informed consent X

# ALLOCATION

**EA**

Small Fibers DPN

X

Sessions 1 to 16

---

Sessions 17 to 32

---

Sham EA

Sessions 1 to 16

---

Sessions 17 to 32

---

Sessions

Axonal DPN

**EA** 1 to 16

---

Sessions 17 to 32

---

Sham EA

Sessions 1 to 16

---

Sessions 17 to 32

---

# ASSESSMENTS:

Demographics X

**Anthropometry** X X X X

**MNSI questionnaire** X X X X

# MDNS

questionnaire X

X X X

**DN-4 questionnaire** X X X X

**NRS questionnaire** X X X X

**SF-36 questionnaire** X X X X

Nerve Conduction

Velocity Study X

X X X

Biochemical profile X

**Inflammation** X X X X

**Oxidative Stress** X X X X

**Genetic expression** X X X X

Treatment adherence

X X X X


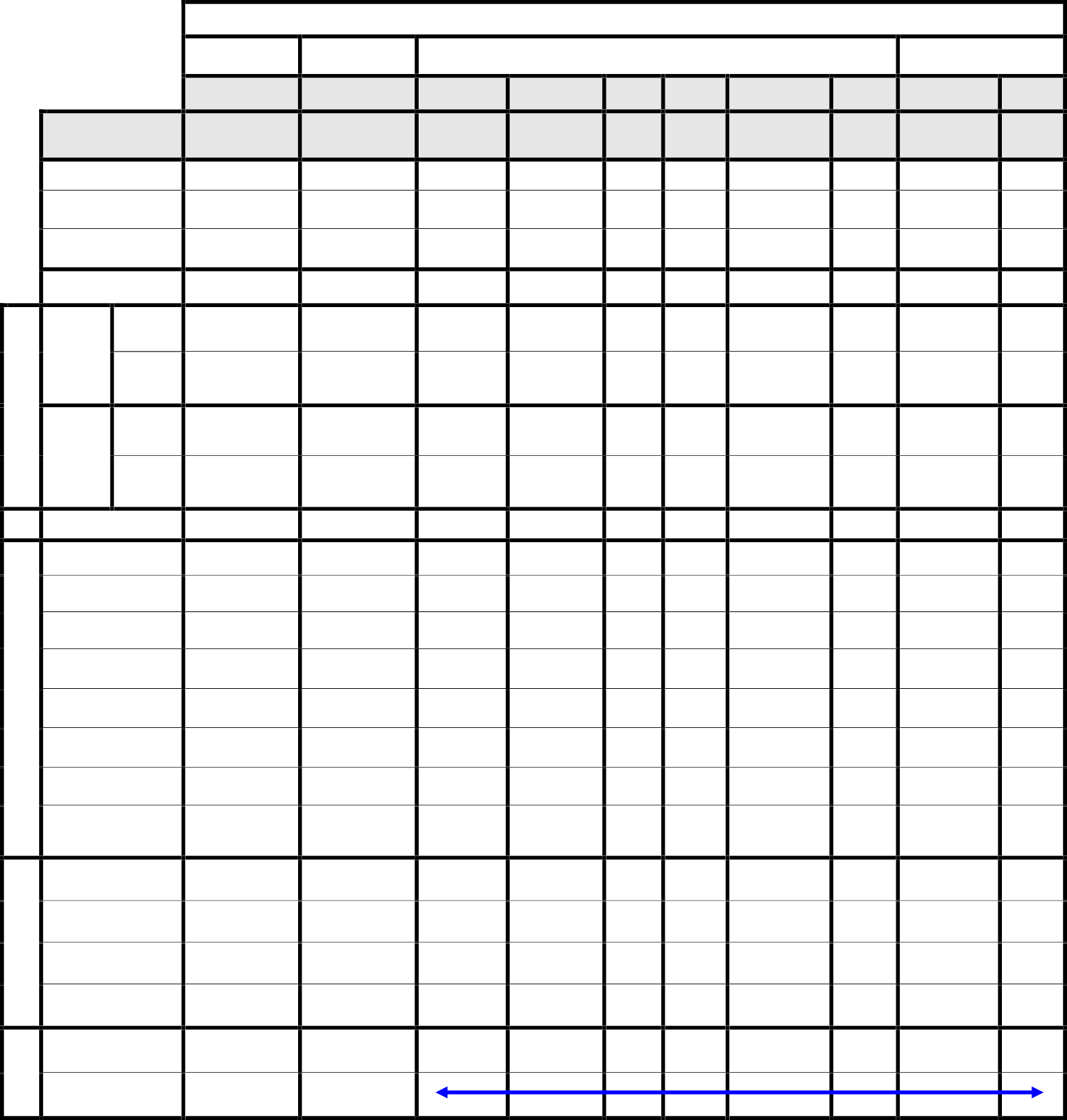


**BIOCHEMICAL & MOLECULAR**

**SECURITY**

**CLINICAL AND ELECTROPHYSIOLOGICAL**

**INTERVENTIONS**

Adverse effects

t0 = Time zero. DPN = Diabetic Polyneuropathy. EA= Electroacupuncture.
