# Supplementary material for: Electroacupuncture efficacy in diabetic polyneuropathy: Study protocol for a double-blinded randomized controlled multicenter clinical trial
Source: BMC Complement Med Ther. 2024 Feb 15;24:90. doi: 10.1186/s12906-024-04375-8 (PMC10868023; doi:10.1186/s12906-024-04375-8)
Supplement: Supplementary file 3 — Additional file 3. Informed Consent form. [file 12906_2024_4375_MOESM3_ESM.docx]

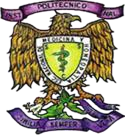

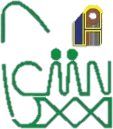
**MEXICAN SOCIAL SECURITY INSTITUTE EDUCATION, RESEARCH AND HEALTH POLICY UNIT HEALTH RESEARCH COORDINATION**

**HIGH SPECIALTY MEDICAL UNIT (UMAE) "DR. BERNARDO SEPÚLVEDA" CENTRO MÉDICO NACIONAL SIGLO XXI, IMSS, CDMX.**

**MEDICAL RESEARCH UNIT IN BIOCHEMISTRY. DEPARTMENT OF NEUROLOGY AND NEUROPHYSIOLOGY.**

**FAMILY MEDICINE UNITS NUMBER 20, 41 AND 44, IMSS, CDMX.**

***SPECIALTY OF HUMAN ACUPUNCTURE* OF THE ENMH OF THE IPN. INFORMED CONSENT LETTER FOR PARTICIPATION IN RESEARCH PROTOCOLS**

Study Name:

"EVALUATION OF THE EFFECT OF ELECTROACUPUNCTURE ON THE SENSORY SYMPTOMS OF SYMMETRICAL DISTAL POLYNEUROPATHY OF DIABETIC ORIGIN AND ITS CORRELATION WITH CHANGES IN NERVE CONDUCTION VELOCITY".

| **External sponsor (if applicable):** | **Not applicable.** |
| --- | --- |
| **Place and date:** | **Mexico City to of the month of of _** _ |
| **Registration number:** | **2020-785-070**, and approval by **CONBIOÉTICA-09-CEI-009-20160601**. |
| **Justification and objective of th study:** | Dear participant it is important to explain that Diabetic Neuropathy is a chronic complication of diabetes and its treatment is usually difficult, there are few treatments for this complication and the success to achieve a total reversal of symptoms so far has been low.  The objective of the present study is to evaluate the effect of electroacupuncture on the sensory symptoms of diabetic neuropathy. Electroacupuncture is a form of acupuncture where a small electric current is passed between pairs of acupuncture needles, this type of approach in different studies has shown benefits in the treatment of diabetic neuropathy, however, researchers have suggested further studies to confirm the findings. To demonstrate the effect of the use of electroacupuncture will be compared with another therapy recognized by Traditional Chinese Medicine called auriculotherapy, which has also been used for the treatment of both chronic diseases, in which millet seeds will adhere to specific regions of the outer ear (ear) without having puncture or electric current and in electroacupuncture.  It is important to mention that since you are a beneficiary of the IMSS, you will also receive as part of this study care and treatment that the family doctor of your clinic considers for the care of diabetes and neuropathy.  You should consider that the participating and responsible for the study are exclusively the Medical Research Unit in Biochemistry, the Department of Neurophysiology being both located in the UMAE "Dr. Bernardo Sepúlveda of the National Medical Center Siglo XXI of the Mexican Institute of Social Security (IMSS), the Family Medicine Units (UMF) number 20, 41 and 44 of the IMSS CDMX and the Human Acupuncture Clinic of the National School of Medicine and Homeopathy of the Institute National Polytechnic (IPN).  It is important that you read in detail the following information which consists of the study procedures explained phases, risks and benefits, in case you wish to participate you must sign this letter of Informed Consent and yo will be indicated the dates and places for the start of the procedure. |
| **Procedures:** | Once the letter of acceptance is signed, a series of questionnaires, physical examination and laboratory and cabinet studies will be carried out to confirm your participation, this is because certain criteria must be met to continue in your participation. First you will be applied a medical history, in which a history of mainly inflammatory and chronic diseases will be asked. Subsequently a series of questionnaires called MDNS, MNSI and NRS that in turn require a physical and clinical examination with the intention of identifying together the presence of signs and symptoms associated with diabetic neuropathy, in addition, a questionnaire will be applied to measure your quality of life called SF36, it is important to mention that the application of these questionnaires and the examination will have an approximate duration of 30 min.  Exclusively the doctor who performed the above evaluation will determine if you can continue with your participation, if so, you will be assigned an appointment at your corresponding UMF with the intention of performing laboratory studies with prior fasting for 8 to 10 hours, this study consists of the determination of blood concentrations of glucose, urea, creatinine, uric acid, lipid profile including triglycerides, total cholesterol, good and bad cholesterol (HDL and LDL) as well as glycosylated hemoglobin (a study that measures average glucose over the past three months).  The intention of the laboratory studies described is to evaluate your metabolic profile, these will also determine if you continue to be a candidate for the study, if you will present any clinical alteration in your laboratory results you will not be a candidate to continue in the study, however, you will receive conventional or specialized medical attention if required by your family doctor or in your case specific reference will be made issued with the support of the authorities of the Corresponding UMF for your care. On the other hand, if you continue to be a candidate you will be assigned an appointment with a date to carry out cabinet studies that will be explained below.  With the intention of analyzing the type of neuropathy that presents, an exclusive appointment will be given to the Department of Neurophysiology of the Specialty Hospital of the CMN Siglo XXI in which you will be evaluated by a neurologist and a neurophysiologist, you will be given a study of electrophysiological techniques, this is a special study (not usual) which lasts approximately 30 minutes and consists of the placement of electrodes attached to the skin ( surface electrodes) to measure the speed of nerve activity and the intensity of signals moving between two or more points, this study is used by scientists and doctors to study and diagnose alterations of the nervous system, as in the case of diabetic neuropathy, also allows to know in which specific region of the nerve would be present a damage, for the present study allows to identify the place and type of neuropathy that you may present, it is important to comment that only people with the diagnosis of  symmetrical distal polyneuropathy will continue, if you will present any alteration or clinical finding that prevents |

|  | your participation in the study, you will receive conventional or specialized medical attention exclusively by the family doctor or in your case by the neurologist or neurophysiologist who has evaluated you, who is responsible for the project. On the other hand, if you continue to be a candidate, you will be assigned a dated appointment to receive care in a traditional Chinese medicine group previously described.  Prior to assigning the date of care, you will be randomly assigned (by lottery) an intervention group with Traditional Chinese Medicine therapy, that is, electroacupuncture or auriculotherapy.  The intervention with Traditional Chinese Medicine will last two months, that is, two sessions per week, that is, 16 sessions in total, each with a time period of 20 minutes, these sessions will be carried out exclusively by doctors specialized in acupuncture in the offices of the National School of Medicine and Homeopathy of the IPN.  After the completion of the two-month intervention, the questionnaires, the biochemical studies, and the electrophysiological technique study will be applied again to analyze whether or not the intervention was successful, the results will be delivered and you will be told which of the two TCM therapeutics was more effective,  In order to evaluate the intervention over time you will be cited three months after the end of the intervention, with the intention of re-evaluating the questionnaires, biochemical studies and electromyography study, this will give us information of which was the most successful over time.  Remember that your family doctor may indicate during your participation a type of conventional treatment, that i general hygienic - dietary measures and could even include pharmacological medication, which should continu as indicated, therefore, you can assign appointments or treatment independently of those stipulated to the stud and that may be important for your treatment. This type of indication will be reported by your family doctor to th responsible researchers for evaluation, it will not affect in any way your participation or services provided in th study. |
| --- | --- |
| **Possible risks and discomforts:** | The present study has a risk greater than the minimum supported by art. 17 and 17 bis of the General Health Law where the risks to health are evaluated, this risk is due to the fact that during the study needles were used for different procedures such as in the extraction of blood, in the study of electromyography and in the application of electroacupuncture.  It is important to note that in each puncture to obtain a blood sample for determination of biochemical parameters will be in a total of 8ml (two tubes of 4 ml each) and will be obtained from the fold of one of the arms, this amount of blood corresponds to almost a "dessert spoonful" (which contains almost 10 ml) this amount will not affect your health at all.  The placement of electrodes attached to the skin of the nerve conduction velocity study, remembering that this is an important study to evaluate the existence of an alteration or damage in the electrical conduction of the nerves (analyzed by this Nerve Conduction Velocity), will be applied in the extremities because there is mainly the symptoms of diabetic neuropathy, and also, although the electrodes are usually safe and not very risky, it is important to tell you that frequencies with electrical voltage are used, and there may be risks that are described below.  The electrode can cause by itself, pain at the site of placement, electrical stimulation of the electrophysiological technique can usually generate neuritis (inflammation of the nerve).  Among the less common but more serious risks are the following: burns, dermal injuries, syncope (dizziness), abscess, and rupture of the electroacupuncture needle. It is important to comment that the alternating frequency used in electroacupuncture is 2/11 hz, the points will be applied in different parts of the body with a duration of 20 minutes per session, the electrical stimulation and its frequency does not involve a risk to your health, however, the discomforts and risks previously mentioned may occur.  We must clarify that the needles used during the study will be sterile, new and disposable, it will be shown before the packaging is used, to verify that it meets these characteristics.  On the other hand, auriculotherapy, consists of the application of millet seeds or steel microspheres applied to the outer ear fixed by means of Micropore, this therapy does not represent a risk of puncture or electrostimulation. All the activities described will be carried out by medical personnel with experience in each area. If you present any discomfort, risk or any of the problems mentioned, you will be attended by the doctor who attends you at that time or in your case you will be referred to the corresponding area.  Note: The UMF, the clinical acupuncture service and the departments of neurology and neurophysiology have the human resources and areas to address the risks or discomfort if required.  Possible benefits you will receive when participating in the study: This study may provide clinical and scientific information for the approach to neuropathic pain in the affected or at-risk population. You will not receive any benefit of an economic, physical or preferential care in the treatment outside the research protocol, on the other hand, the complete clinical evaluation, the stipulated electroacupuncture or auriculotherapy service, the delivery and explanation of the laboratory and cabinet studies, as well as the medical attention you will receive in the study will be at no cost, making the clarification that includes only those stipulated in the project. This study with personalized attention can help guide you to a better treatment, a timely diagnosis and generate prevention measures in the future towards complications. Participating will not grant you any other benefits outside of the stipulations in any of the participating places. As mentioned above, if you present any alteration at the beginning or in the course of the study derived from it, it will be supported with the necessary attention, hence also the importance of inviting only the right holders.  Information on results and treatment alternatives: At the end of the study you will be made known personally an confidentially the results, in addition, you will be invited to continue with the treatment that is most benefici according to the results of the study. The use of Traditional Chinese Medicine is considered as an alternativ treatment of diabetic neuropathy, in this study as explained the intention is to know the effectiveness of the use electroacupuncture which will be compared with another therapy recognized by Traditional Chinese Medicin auriculotherapy. Different researchers in the field have recommended conducting more research to have mor scientific evidence of the use of electroacupuncture hence the importance of this study. |
| **Participation or withdrawal:** | Your participation will be voluntary, therefore, despite having signed this letter of informed consent, you at the tim you decide not to continue may do so, without interfering with the care you receive in your corresponding fami medicine unit. |
| **Privacy and confidentiality:** | Strict confidentiality of the results will be kept and used only for the purposes of the research n.n. |

| **In case of collection of biologic**  **material (if applicable) I marked th following table:** | **I DO authorize the sample to be taken.** |
| --- | --- |
| **Availability of medical treatment f beneficiaries:** | Not applicable. |
| **Benefits at the end of the study:** | To know if the use of electroacupuncture was effective in relieving the symptomatology of diabetic neuropath when compared with another classic therapeutic intervention of Traditional Chinese Medicine, in addition, t evaluate if it led to a better quality of life evaluated with validated instruments. All this information will give a important contribution of scientific knowledge to support you and support other people in the future and th approach to diabetic neuropathy. |
| **Main Researcher:** | In case of doubts or clarifications all will be answered and clarified by the doctors participating in the study, bein the main responsible Dr. JOSÉ DE JESÚS PERALTA ROMERO. Doctor and Researcher responsible for th project, Matricula 311090812, Office: Medical Research Unit in Biochemistry 1st Floor, Specialty Hospital, Avenid Cuauhtémoc 330, Colonia Doctores. Mexico City, CP 06720. CMN Siglo XXI, IMSS. Tels IMSS 56276900 EX 21477, Office: 5519 6193 Cel 04455 3231 8563, from 9 a.m. to 4 p.m. from Monday to Friday or by ema [drjperalta@hotmail.com](mailto:drjperalta@hotmail.com) |
| **In case of DOUBTS O CLARIFICATIONS**  **ABOUT YOUR RIGHTS AS**  **PARTICIPANT may contact:** | **IMSS RESEARCH ETHICS COMMITTEE: Avenida Cuauhtémoc 330 4th floor Block "B" of the Congres Unit, Colonia Doctores. Mexico City, CP 06720. Phone (55) 56276900 extension 21216 from 9 a.m. to 4 p.m monday through Friday, Email:** [**comiteeticainv.imss@gmail.com**](mailto:comiteeticainv.imss@gmail.com) |

If you agree to place below full name, address, relationship, telephone number and signature:

NAME/ADDRESS/SIGNATURE OF THE PATIENT NAME/ADDRESS/SIGNATURE

OF THE PACINETE FROM WHOM THE CONSENT IS OBTAINED

NAME/ RELATIONSHIP/ ADDRESS / SIGNATURE NAME/ RELATIONSHIP/ ADDRESS / SIGNATURE

This format constitutes a guide that must be completed according to the characteristics of each research protocol, without omitting relevant information from the study.

**Property ID: 2810-009-013**
