# Supplementary material for: Electroacupuncture efficacy in diabetic polyneuropathy: Study protocol for a double-blinded randomized controlled multicenter clinical trial
Source: BMC Complement Med Ther. 2024 Feb 15;24:90. doi: 10.1186/s12906-024-04375-8 (PMC10868023; doi:10.1186/s12906-024-04375-8)
Supplement: Supplementary file 4 — Additional file 4: Supplementary figure 2. protocol consort diagram. [file 12906_2024_4375_MOESM4_ESM.docx]

SUPLEMENTARY FIGURE 2. PROTOCOL CONSORT DIAGRAM

**INTERVENTION OF FIVE MONTHS**

**INTERVENTION OF FIVE MONTHS**

**ALLOCATION**

**ALLOCATION**

**Intervention in the Department of Acupuncture of the Escuela Nacional de Medicina y Homeopatía, Instituto Politécnico Nacional**

**Unidad Médica de Alta Especialidad**

**"Dr. Bernardo Sepúlveda" Centro Médico Nacional Siglo XXI**

**Instituto Mexicano del Seguro Social**

**STRATIFICATION**

**(N=40 patients)**

**Axonal DPN (n=20)**

- Sensory and motor neuropathic symptoms.
- Decreased tendon reflexes and muscle strength.
- NCS, amplitude, and decreased potentials, blocks in 5 nerves.

**Short fibers DPN (n=20)**

- Sensory neuropathic symptoms.
- Preserved or diminished osteotendinous reflexes.
- NCS unchanged.
- Determination of baseline parameters*.
- Clinical diagnosis of DPN by Neurology department.
- Nerve Conduction Study (NCS) by Neurophysiology department.
- Unidad de Investigación Médica en Bioquímica.

**SCREENING**

- Medical history asessment.
- Physical examination, focused on Diabetic Polyneuropathy (DPN).
- Application of MNSI, MDNS, DN-4, NRS, SF-36 questionnaires and therapeutic adherence.

**Unidad de Medicina Familiar 20,41 and 44**

- Search of patients in SIM system database of participating UMF.
- Application of TRIAGE of **SELECTION CRITERIA,** via telephone:

o Age 40 to 75 years.

o Type 2 diabetes mellitus f rom 5 to 20 years of evolution.

o Body Mass Index (BMI) 18 to 35 kg/m2..

o Glycosylated hemoglobin (HbA1c%) ≥ 7%.

o Sensory or motor symptoms of diabetic polyneuropathy (DPN).

o No history of immunodeficiencies, autoimmune diseases, hip or spine f racture, acute neuropathies or other than PNDS.

**RECRUITMENT**

**ANALYSIS OF RESULTS AT THE END OF THE INTERVENTION. *≠**

**Electroacupuncture (n= 10)**

- 32 EA sessions in total.
- 2 intervention cycles of two months each.
- One month of rest between each cycle (tachyphylaxis).
- 16 sessions per cycle.
- 2 sessions per week.
- 20 minutes each session.
- Electroacupuncture at an alternate frequency (2 to 100 Hz).

**EVALUATION OF EFFECTIVENESS OVER TIME THREE MONTHS AFTER THE INTERVENTION. * ≠**

**Sham electroacupuncture (n= 10)**

- 32 sessions of fake electroacupuncture in total.
- 2 intervention cycles of two months each.
- One month of rest between each cycle.
- 16 sessions per cycle.
- 2 sessions per week.
- 20 minutes each session.
- Sham EA device (nonpuncture and nonelectrical stimulus).

**Sham electroacupuncture (n= 10)**

- 32 sessions of fake electroacupuncture in total.
- 2 intervention cycles of two months each.
- One month of rest between each cycle.
- 16 sessions per cycle.
- 2 sessions per week.
- 20 minutes each session.
- Sham EA device (nonpuncture and nonelectrical stimulus).

**Electroacupuncture (n= 10)**

- 32 EA sessions in total.
- 2 intervention cycles of two months each.
- One month of rest between each cycle (tachyphylaxis).
- 16 sessions per cycle.
- 2 sessions per week.
- 20 minutes each session.
- Electroacupuncture at an alternate frequency (2 to 100 Hz).

*Determination of anthropometry (weight, height, BMI), blood pressure, biochemical (glucose, urea, creatinine, total cholesterol, HbA1c %), and molecular parameters (cytokines, oxidative stress, and gene expression), at the end of the intervention in its corresponding family care unit.

≠ Application of MNDS, MNSI, DN-4 NRS, SF-36 questionnaires and therapeutic adherence.

≠ Directed physical examination of the nervous system at the end of the intervention, by the neurology service.

≠NCS by neurophysiology service at the end of the intervention.
